# Expression of Concern: MicroRNA-34A inhibits the growth, invasion and metastasis of gastric cancer by targeting PDGFR and MET expression

**DOI:** 10.1042/BSR-20140020_EOC

**Published:** 2021-04-06

**Keywords:** gastric cancer, MET, miR-34a, PDGFR

The Editorial Office has been made aware of potential issues surrounding the scientific validity of this paper, hence has issued an expression of concern to notify readers whilst the Editorial Office investigates.

